# Whole genome sequencing confirmed the *de novo* development of macrolide resistance in a child with *Mycoplasma pneumoniae* infection receiving azithromycin treatment

**DOI:** 10.1128/asmcr.00088-25

**Published:** 2025-07-02

**Authors:** Huanyu Wang, Sophonie J. Oyeniran, Kathy Everhart, Amy L. Leber

**Affiliations:** 1 Department of Pathology and Laboratory Medicine, Nationwide Children’s Hospital, Columbus, Ohio, USA; 2 Department of Pathology, The Ohio State University 2647, Columbus, Ohio, USA; 3 Department of Pediatrics, Division of Infectious Diseases, Nationwide Children’s Hospital and The Ohio State University 2647, Columbus, Ohio, USA; Rush University Medical Center, Chicago, Illinois, USA

**Keywords:** *Mycoplasma pneumoniae*, macrolide resistance, whole genome sequencing

## Abstract

**Background:**

*Mycoplasma pneumoniae* (MPN) is a major pathogen of community-acquired respiratory tract infections, and macrolides are the drug of choice for treating MPN infections. Macrolide resistance in our patient population has been low; however, an increase in resistance rates was observed during a recent resurgence.

**Case Summary:**

A previously healthy 11-year-old male was diagnosed with MPN infection and received azithromycin treatment. His symptoms continued after two courses of azithromycin, and he tested positive for MPN DNA three times. Sanger sequencing of samples collected after his first course of azithromycin revealed a mutation conferring macrolide resistance, while the sample collected before the initiation of antibiotics did not. Additionally, whole genome sequencing (WGS) and single-nucleotide polymorphism (SNP) analysis demonstrated that samples collected before and after receiving azithromycin differed by one SNP, indicating that the two isolates are the same strain, and the development of macrolide resistance occurred *de novo* during treatment.

**Conclusion:**

Our case utilized WGS to confirm that the development of *de novo* macrolide resistance can occur rapidly following macrolide use. Clinicians should be vigilant for macrolide treatment failure and consider alternative drugs if symptoms persist or there are signs of clinical deterioration.

## INTRODUCTION

*Mycoplasma pneumoniae* (MPN) is a major pathogen of community-acquired respiratory tract infections that causes epidemics every few years. The non-pharmaceutical interventions in place during the COVID-19 pandemic interrupted the epidemics of MPN and other respiratory pathogens. Since September 2023, MPN re-emerged in our patient population, resulting in the largest outbreak seen in central Ohio in which more than 7,000 children were infected (1).

Macrolides are the drug of choice for treating MPN infections. The resistance of MPN to macrolides is conferred by point mutations within the V region of 23S rRNA, of which the change of A to G at location 2063 of the gene (A2063G) is the most common, followed by A2064G. While Eastern Asian countries report macrolide resistance in >90% MPN strains (2), resistance rates remain low in USA and Western European countries (1, 3). We recently reported an average of 2.4% macrolide-resistant MPN (MRMp) in our patient population post-COVID-19 pandemic (1); however, resistance rates increased over time, with the highest rate, over 8%, observed in October 2024 (unpublished data). Here, we report a case with documented development of MRMp in a patient receiving azithromycin using whole genome sequencing (WGS).

## CASE PRESENTATION

A previously healthy 11-year-old male presented to his primary care physician (PCP) with the chief complaint of cough (Table 1, day 1). Congestion started a week prior to presentation and cough 4 days prior. He denied fever, body ache, or sore throat. Physical examination was normal. According to PCP notes, symptoms were consistent with a viral upper respiratory tract infection. However, due to the increased activity of MPN in the community, MPN PCR was ordered and found to be positive. The patient received a 5-day course of azithromycin. Fourteen days after this visit, he presented again with an ongoing cough, which was described as “wet sounding” (Table 1, day 14). His symptoms had improved slightly within the first few days of antibiotics but never fully resolved. MPN PCR was ordered and tested positive again. He was given another 5-day course of azithromycin and sent home. Two days after finishing azithromycin treatment, the patient presented to his PCP without significant improvement, although no new symptoms (Table 1, day 20). MPN was detected again from a third collection, and doxycycline was prescribed for 10 days. No testing other than MPN PCR was ordered during these encounters.

**TABLE 1:** Clinical and laboratory findings for patient with *Mycoplasma pneumoniae* infection^*a*^

	Day 1^*b*^	Day 14	Day 20
Symptoms	Cough, congestion	Cough, “wet sounding”	Cough
SOC testing specimen type	Throat swab	Throat swab	Throat swab
SOC testing result	Positive Ct 29.5	Positive Ct 27.9	Positive Ct 35.3
Antibiotics prescribed	Azithromycin 5 days	Azithromycin 5 days	Doxycycline 10 days
Sanger sequencing of partial 23S rRNA	No mutation detected	A2063G mutation	Not done
MPN culture	Positive	Positive	Negative
Whole genome sequencing and phylogenetic analysis	No mutation detected Fig. 1: NCH MPN_1	A2063G mutation Fig. 1: NCH MPN_2	NA
MLST	ST20	ST20	NA

^*a*^ SOC: standard of care; MPN: *Mycoplasma pneumonia;* Ct: cycle threshold; NA: not available; MLST: multi-locus sequencing type.

^*b*^ Days are numbered from the first tested positive for *M. pneumoniae.*

His persistent symptoms and lack of response to azithromycin raised concern for macrolide resistance. Samples from the first and second medical encounters were retrieved, and Sanger sequencing of the partial 23S rRNA gene was performed on the primary samples for the detection of common mutations conferring macrolide resistance (1). We attempted to culture all samples but were successful only with the first two (4); both cultured isolates were sent for WGS (CosmosID, Germantown, MD). The sequences of these two samples, along with genome data of MPN strains from other geographic locations and years that were retrieved from the NCBI database, were analyzed using an online tool for single-nucleotide polymorphism (SNP) identification and phylogeny analysis (Center for Genomic Epidemiology, CSIPhylogeny v 1.4). A phylogenetic tree was visualized with MEGA11 software (Molecular Evolutionary Genetics Analysis version 11). Multi-locus sequencing type (MLST) of these samples was determined by comparing the genome sequences to the *Mycoplasma pneumoniae* typing database (https://pubmlst.org/organisms/mycoplasma-pneumoniae).

**Fig 1:**
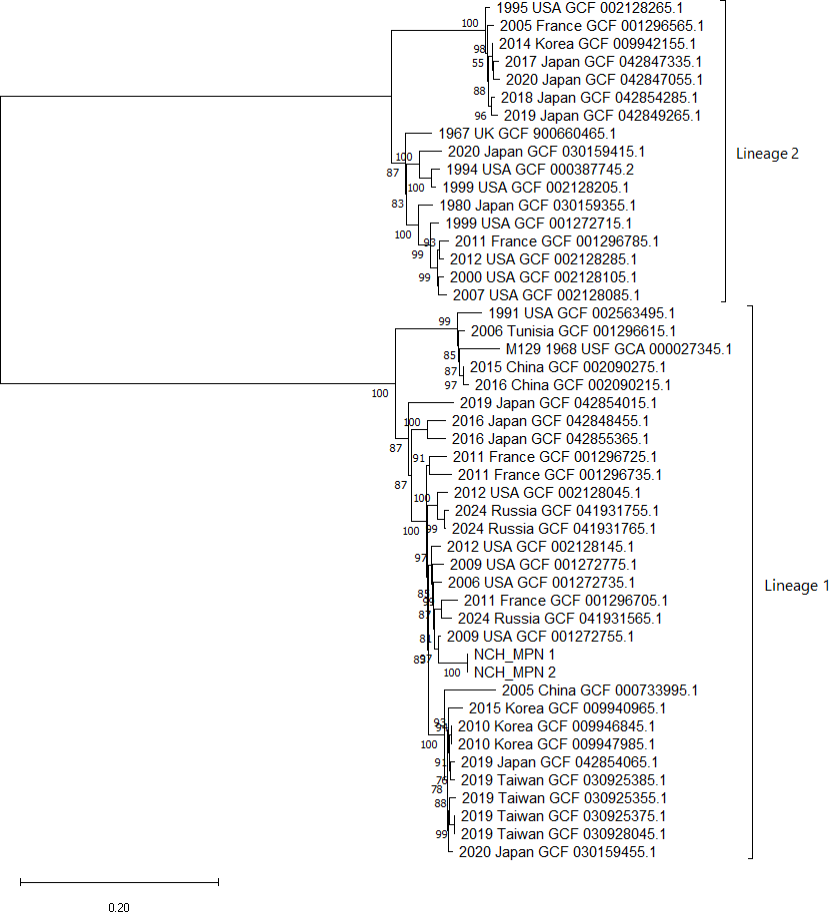
SNP phylogenetic tree generated using an online bioinformatics tool (Center for Genomic Epidemiology, CSIPhylogene) and visualized with MEGA v11. Sequences are labeled with the locations and the years of isolation, as well as RefSeq Assembly Numbers.

Sanger sequencing of the primary samples revealed an A2063G mutation from the sample collected at the second medical encounter (day 14, Table 1) but not the sample collected on day 1. The presence of the A2063G mutation in the second sample and the absence of any mutation that confers macrolide resistance in the first sample were confirmed by WGS. SNP phylogenetic analysis showed that the two isolates (NCH MPN_1 and NCH MPN_2) are clustered together and are most closely related to a strain collected in the USA in 2009 (GCF: 001272755) (Fig. 1). All three clustered with the type strain M129 of type 1 (*p1* typing) lineage (GCF: 000027345.1) and were separate from the type 2 lineage cluster. The two samples from this patient differed by one SNP, suggesting the strains are identical and that the development of macrolide resistance occurred *de novo* following azithromycin treatment. Both samples’ MLST type was ST20.

## DISCUSSION

Prior macrolide exposure is associated with the development of macrolide resistance (5). Unlike other bacteria, MPN has a single rRNA operon, and therefore, a single mutational event can lead to macrolide resistance. It has been shown that resistance can develop *in vitro* by incubating and sub-culturing MPN strains in sub-inhibitory concentrations of macrolides (6), and cases of macrolide resistance developing in patients who underwent treatment have been reported previously (7–12). In addition, WGS and phylogenetic analysis of strains from different geographic locations and years have found that strains of similar origins clustered together and macrolide-resistant strains were present in most of the clusters; these data support that MRMp evolved independently, as a result of the use of macrolides, from various genetic backgrounds (13).

The MPN genome is extraordinarily stable over time, and strains with geographic distances across the globe are >99% identical to each other (14). In various case studies reporting macrolide resistance developing during treatment, multiple typing schemes were used: Sanger sequencing of the V region of 23S rRNA, analysis of the gene encoding the adhesin P1, multi-locus variable-number tandem repeat analysis (MLVA), and MLST or SNP typing (7–11). Each of these typing methods has its limitations; for example, Averbuch et al. described a previously healthy boy with MPN infection and macrolide resistance developed during treatment. Sanger sequencing demonstrated a mixed population of a wild-type strain and a strain carrying an A2063G mutation (15), suggesting the possibility that a mixed population existed at the time of infection and that macrolide resistance was a result of selection of the resistant strain already present prior to macrolide exposure. Another case reported the emergence of two mutations on the 23S rRNA gene after completion of macrolide treatment in a patient with a complex medical history. MLVA showed that the resistant strain and wild-type strain had the same MLVA type; the authors concluded that the resistant strain evolved from the wild-type strain (10). However, a recent study utilizing WGS combined with SNP analysis revealed that strains within the same MLVA type are not necessarily identical or share similar genetic backgrounds (13). Therefore, MLVA typing may not provide sufficient evidence to confirm that strains are the same in this case. In our study, we sequenced the entire genome and performed WGS-SNP analysis, which provided a much more in-depth analysis of the sequences compared to the typing methods as described above. Our WGS-SNP analysis demonstrated that the patient’s two isolates differed by one SNP and confirmed that the A2063G mutation indeed occurred *de novo* following macrolide exposure.

Macrolides remain the first-line drug for MPN infection. Fluoroquinolones and tetracyclines, although generally not recommended in children due to possible side effects, are an alternative when there is concern for MPMp. A2063G and A2064G account for >99% of reported mutations conferring macrolide resistance in MPN, and isolates carrying these mutations demonstrate high minimum inhibitory concentrations against macrolides (e.g., 256 mg/mL for azithromycin). However, macrolides still appeared to be clinically effective in some patients infected with MRMp, albeit with lower efficacy (16, 17). This could be explained by the fact that MPN infections are often self-limited, as well as macrolide’s anti-inflammatory effects and immunomodulatory potential that may contribute to improved clinical symptoms (18). Randomized controlled studies have also demonstrated the benefit of combined treatment with macrolides and another antibiotic in cases with severe community acquired pneumonia (CAP), which was possibly due to macrolides’ anti-inflammatory activities rather than anti-microbial activities (18). In our case, despite the patient’s receipt of a 5-day course of azithromycin, the cycle threshold (Ct) value obtained from day 14 was similar to that of day 1, demonstrating the persistence of bacteria at the site of detection and treatment failure, consistent with the development of macrolide resistance. Interestingly, after two courses of azithromycin treatment and before the initiation of doxycycline, the Ct value of samples increased from 27 to 35 (Table 1, day 14 vs day 20), and the culture of the sample collected on day 20 failed to grow. This reduction of bacterial burden suggests that the positive result from day 20 may be due to the detection of bacterial DNA or non-viable organisms and may indicate that the infection was responding to therapy even without targeted antibiotics.

Our case supports that the development of macrolide resistance in MPN is related to macrolide administration and occurs *de novo*. Use of WGS allowed a more accurate assessment of this compared to other typing methods. While our patient was not severely ill and may have recovered without the use of an alternative agent, clinicians should be vigilant for macrolide treatment failure and consider the use of alternative treatment regimens if symptoms persist or there are signs of clinical deterioration, particularly in severely ill patients. This study highlights the importance of rapid diagnostic assays to detect macrolide resistance, especially in regions where MRMp rates are low, and supports repeat testing in patients whose symptoms persist while on appropriate therapy, as macrolide resistance can develop rapidly.

## Data Availability

The genomic sequences of the isolates were submitted to the NCBI GenBank under BioSample accessions SAMN48337020 and SAMN48337083.
